# Novel Mechanism for Memantine in Attenuating Diabetic Neuropathic Pain in Mice via Downregulating the Spinal HMGB1/TRL4/NF-kB Inflammatory Axis

**DOI:** 10.3390/ph14040307

**Published:** 2021-04-01

**Authors:** Suliman Y. Alomar, Rehab E. Abo El Gheit, Eman T. Enan, Khaled S. El-Bayoumi, Mohamed Z. Shoaeir, Amany Y. Elkazaz, Sultan S. Al Thagfan, Sawsan A. Zaitone, Rehab M. El-Sayed

**Affiliations:** 1 Department of Zoology, College of Science, King Saud University, Riyadh 11495, Saudi Arabia; 2 Department of Physiology, Faculty of Medicine, Tanta University, Tanta 31527, Egypt; rehab.abouelghait@med.TanTa.edu.eg; 3 Department of Pathology, Faculty of Medicine, Mansoura University, Mansoura 35516, Egypt; emanenan@mans.edu.eg; 4 Department of Human Anatomy and Embryology, Faculty of Medicine, Mansoura University, Mansoura 35516, Egypt; khalad200772@mans.edu.eg; 5 Department of Rheumatology and Rehabilitation, Al-Azhar Asyut Faculty of Medicine for Men, Assiut 71524, Egypt; mohamedakaria.2044@azhar.edu.eg; 6 Biochemistry and Molecular Biology Department, Faculty of Medicine, Suez Canal University, Ismailia 41522, Egypt; amany_elkazaz@med.suez.edu.eg; 7 Biochemistry and Molecular Biology Department, Faculty of Medicine, Port-Said University, Port Said 42526, Egypt; 8 Department of Clinical and Hospital Pharmacy, College of Pharmacy, Taibah University, Al Madinah Al Munawwarah 41311, Saudi Arabia; salthagfan@gmail.com; 9 Department of Pharmacology & Toxicology, Faculty of Pharmacy, Suez Canal University, Ismailia 41522, Egypt; 10 Department of Pharmacology & Toxicology, Faculty of Pharmacy, University of Tabuk, Tabuk 71451, Saudi Arabia; 11 Department of Pharmacology & Toxicology, Faculty of Pharmacy, Sinai University, El-Arish, North Sinai 45511, Egypt; rehab.mahmoud@su.edu.eg

**Keywords:** glutamate, HMGB1/TRL4/NF-kB axis, memantine, mouse diabetic neuropathy, sciatic pathology

## Abstract

Diabetic neuropathic pain (DNP) is a common diabetic complication that currently lacks an efficient therapy. The aim of the current work was to uncover the anti-allodynic and neuroprotective effects of memantine in a model of mouse diabetic neuropathy and its ameliorative effect on the high-mobility group box-1 (HMGB1)/toll-like receptor 4 (TLR4)/nuclear factor-k B (NF-kB) inflammatory axis. Diabetes was prompted by an alloxan injection (180 mg/kg) to albino mice. On the ninth week after diabetes induction, DNP was confirmed. Diabetic mice were randomly allocated to two groups (six mice each); a diabetes mellitus (DM) group and DM+memantine group (10 mg/kg, daily) for five weeks. DNP-related behaviors were assessed in terms of thermal hyperalgesia and mechanical allodynia by hot-plate and von Frey filaments. Enzyme-linked immunosorbent assay (ELISA) kits were used to measure the spinal glutamate, interleukin-1 beta (IL-1β), and tumor necrosis factor-α (TNF-α). The spinal levels of N-methyl-D-aspartate type 1 receptor (NMDAR1), HMGB1, TLR4, and phosphorylated NF-kB were assessed using Western blotting. Histopathological investigation of the spinal cord and sciatic nerves, together with the spinal cord ultrastructure, was employed for assessment of the neuroprotective effect. Memantine alleviated pain indicators in diabetic mice and suppressed excessive NMDAR1 activation, glutamate, and pro-inflammatory cytokine release in the spinal cord. The current study validated the ability of memantine to combat the HMGB1/TLR4/NF-kB axis and modulate overactive glutamate spinal transmission, corroborating memantine as an appealing therapeutic target in DNP.

## 1. Introduction

Diabetic neuropathic pain (DNP) is an insufferable, long term, and precise category of permanent pain. It originates as a direct result of pathological alterations disturbing the somatosensory system [1,2] as an outcome of the metabolic disturbances in diabetes [3]. Clinically, DNP can be elicited by non-painful stimuli (allodyniA) or felt in an exaggerated manner in response to noxious stimuli (hyperalgesiA) [4].

Although the pathogenesis of DNP has been extensively investigated, the underlying molecular mechanisms remain to be explored. In spite of its chronic nature and the disrupted homeostatic perception and processing of DNP, there are no effective therapies, which adds misery to the already crippled patient’s life [5]. Despite the fact that that hyperglycemia, autonomic dysfunction, and increased thalamic vascularity were proposed to be mechanistic contributors in DNP [6], recent evidence pointed to the prevalence of abnormalities in the central and peripheral pain pathways [5].

High-mobility group box 1 (HMGB1) is a very conserved ubiquitous nuclear protein. Under physiological conditions, it acts as a DNA chaperone within the nucleus. Its release takes place either passively by necrotic cells or actively from the activated inflammatory cells or stimulated neurons [7]. Once located extracellularly, HMGB1 amplifies proinflammatory and pronociceptive signals through its action on the innate immune receptors comprising toll-like receptors (TLR2 and TLR4) [7,8]. Indeed, activation of the HMGB1-TLR4 axis upregulates phosphorylated nuclear factor-kappa B (p-NF-kB), resulting in the enhanced expression and release of the inflammatory markers—interlukin-1β (IL-1β) and tumor necrosis factor-α (TNF-α) [7]. Additionally, N-methyl D-aspartate (NMDA) receptors contribute to HMGB1-mediated inflammatory response. HMGB1 enhances the activation for NMDA receptor-induced neural damage [9,10] as result of Ca2+ influx and nitric oxide synthase induction leading to cell death [11].

The NMDA receptor antagonist, memantine, displays beneficial effects in Alzheimer’s disease pathology [12]. Recent evidence highlighted the role of memantine in the inflammatory response [13,14] and in formalin-induced behaviors in rats [15]. Many clinical trials encouraged the use of memantine in treating painful DNP [16,17], post-surgical neuropathic pain [18], postherpetic neuralgia [19], and chronic phantom limb pain [20].

However, the precise pharmacologic mechanism for memantine’s neuroprotective potential in DNP has yet to be fully elaborated and, hence, in the current study, we aimed to investigate this point. We investigated the consequences of NMDA receptor inhibition by memantine on the spinal HMGB1/TLR/NF-kB axis in a mouse DNP model.

## 2. Results

**Figure 1:**
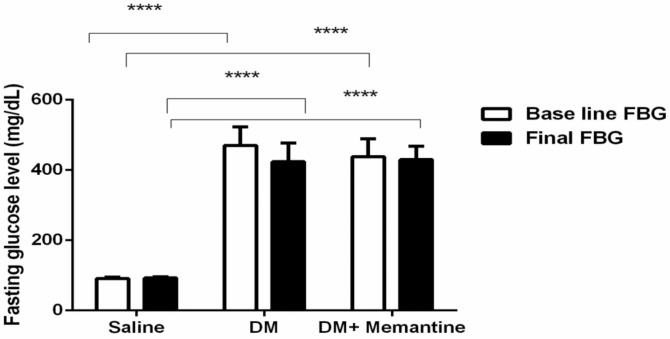
Effect of memantine on the blood glucose level. Fasting blood glucose was measured before memantine therapy and at the end of the experiment. The data are the mean ± standard deviation of the mean (SDM) and were analyzed by one-way ANOVA and Tukey’s tests. DM: diabetes mellitus mice, **** *p* < 0.0001 versus the saline group.

The baseline and final blood glucose results are presented in Figure 1. At the two occasions, the blood glucose levels in the diabetes mellitus (DM) control group and the DM+memantine group were greater than the level registered in the saline group. Importantly, treatment with memantine did not affect the blood glucose level compared to the DM control group (Figure 1).

**Figure 2:**
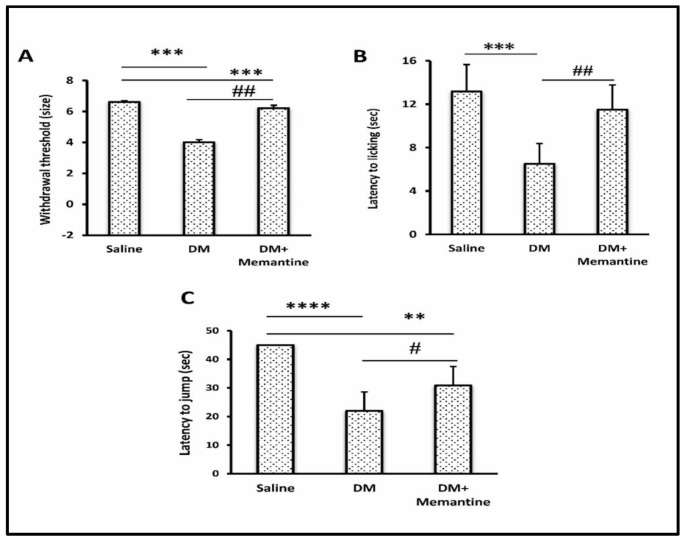
Effect of memantine on allodynia and hyperalgesia. Diabetic mice were tested for (**A**) the von Frey test, (**B**) latency to licking (sec) and (**C**) latency to jump. Data are the mean ± SDM and analyzed by one-way ANOVA and Tukey’s tests. DM: diabetes mellitus mice, SDM: standard deviation of the mean. ** *p* < 0.01, *** *p* < 0.001, and **** *p* < 0.0001, versus the saline group; # *p* < 0.05 and ## *p* < 0.01 versus the DM group.

The results showed that the DM control group acquired lesser thresholds for the mechanical allodynic stimuli versus the saline control mice (Figure 2A) and the thermal hyperalgesic stimuli in the hot plate test (latency to licking 6.5 ± 1.87 s vs. 13.17 ± 2.48 s and latency to jumping 22 ± 6.63 vs. 45 ± 0, Figure 2B,C). The DM+memantine group showed improvements in response to mechanical allodynia (126.67 ± 41.31, Figure 2A) as well as latency to licking (11.5 ± 2.26, Figure 2B) and latency to jumping (30.83 ± 6.62, Figure 2C) in response to thermal hyperalgesia.

Importantly, normal mice treated with memantine (10 mg/kg) did not display a significant alteration from the saline control group in response to mechanical allodynic stimuli (6.58 ± 0.1) or latency to licking (14.5 ± 5.89) as well as latency to jumping (43.5 ± 2.51) as a response to thermal hyperalgesic stimuli (data not shown in illustrations).

**Figure 3:**
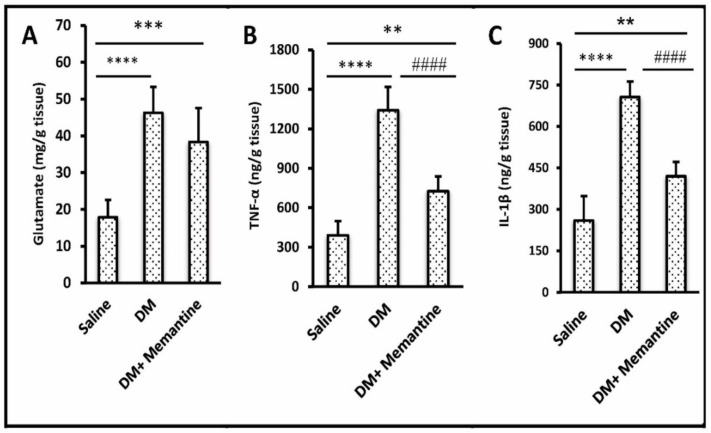
Effect of memantine on spinal glutamate and inflammatory mediators. Spinal cord samples were analyzed for (**A**) glutamate, (**B**) TNF-α, and (**C**) IL-1β. Data are the mean ± SDM and analyzed by one-way ANOVA and Tukey’s tests. DM: diabetes mellitus mice, and SDM: standard deviation of the mean, TNF-α: tumor necrosis factor-α, IL-1β: interlukin-1β. ** *p* < 0.01, *** *p* < 0.001, and **** *p* < 0.0001, versus the saline group; #### *p* < 0.0001 versus the DM group.

Measuring the spinal glutamate level indicated a 2.59-fold increase in the DM group versus the saline group while DM+memantine group did not display a significant alteration in the spinal glutamate content versus the DM group (*p* > 0.05, Figure 3A). The spinal levels of TNF-α and IL-1β were elevated (3.44-fold and 2.73-folD) in the DM group versus the saline control group (Figure 3B,C).

The DM+memantine group showed significant lowering in the spinal level of these two inflammatory cytokines versus the DM group. The saline+memantine group presented a spinal glutamate content (17.26 ± 7.86), TNF-α (407.67 ± 75.43), and IL-1β (275.17 ± 100.27) similar to those detected in the saline control group (data not demonstrated in illustrations). Overall, these results indicate that memantine has no effect, per se, on the spinal markers in saline injected mice.

**Figure 4:**
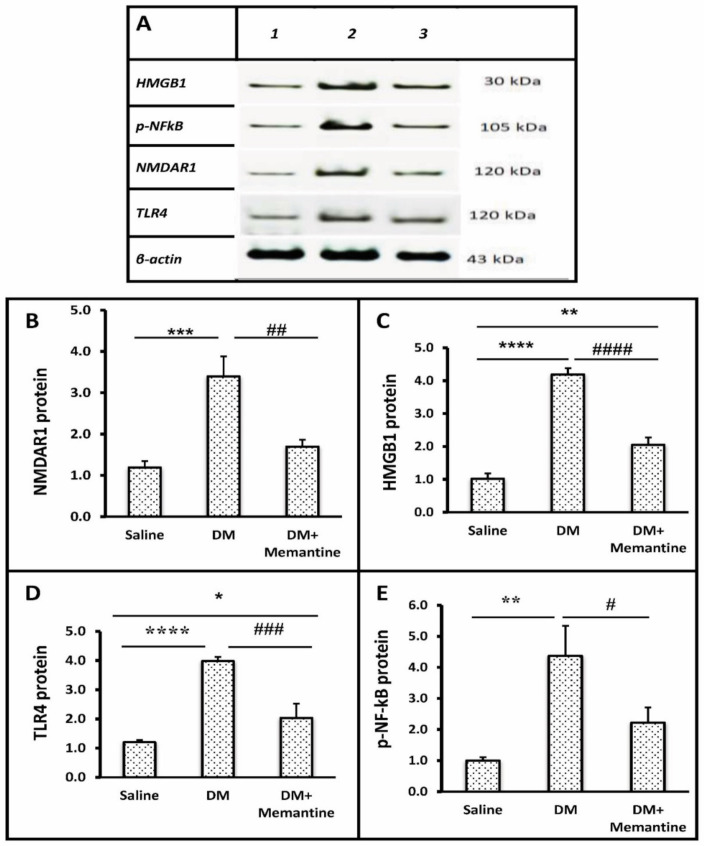
Effect of memantine on the spinal protein level of the inflammatory markers. (**A**) Spinal cord samples from saline (#1), diabetic (#2), and DM+memantine (10 mg/kg) groups (#3) were analyzed for (**B**) NMDAR1, (**C**) HMGB1, (**D**) TLR4 and (**E**) p-NF-kB. Data are mean ± SDM and analyzed by one-way ANOVA and Tukey’s tests. DM: diabetes mellitus mice, NMDAR1: n-methyl-D-aspartate receptors, HMGB1: high-mobility group protein 1, p-NF-kB: phosphorylated nuclear factor-kappa B, TLR4: Toll-like receptor 4, and SDM: standard deviation of the mean. * *p* < 0.05, ** *p* < 0.01, *** *p* < 0.001, and **** *p* < 0.0001 versus the saline group; # *p* < 0.05, ## *p* < 0.01, ### *p* < 0.001, and #### *p* < 0.0001 versus the DM group.

Figure 4 shows the protein content of N-methyl-D-aspartate type 1 receptor (NMDAR1), HMGB1, TLR4, and p-NF-kB versus the β-actin protein. Analysis of the Western blot results highlighted a greater spinal level of the NMDAR1 (2.85-fold, Figure 4A,B), HMGB1 (4.1-fold, Figure 4A,C), TLR4 (3.16-fold, Figure 4A,D), and p-NF-kB (4.39-fold, Figure 4A,E) proteins in the DM group versus the saline group. The DM+memantine group showed a significantly lower content of these spinal markers compared to the DM group (Figure 4B–E).

**Figure 5:**
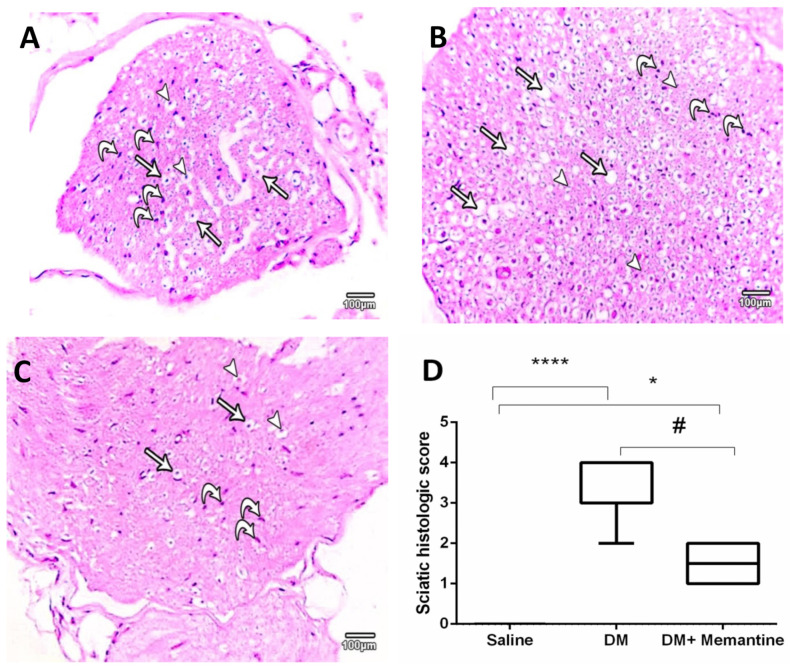
Microscopic pictures of hematoxylin and eosin stained sections of sciatic nerves. (**A**) An image from the saline group presenting multiple axons (arrowheads) found in different thicknesses of myelin sheathes (arrows) and enclosed within the endoneurium with multiple nuclei of Schwann cells (curved arrows). (**B**) Diabetic group (DM) showing several lost axons (arrows) with lost myelin sheathes (arrowheads) with some Schwann cell nuclei (curved arrows). (**C** DM+memantine shows normal neuronal axons (arrowheads) with normal myelin sheath (arrows) and an increased number of Schwann cells nuclei (curved arrows). ×100 bar 100 μm. (**D)**) Data from histopathologic scoring of the sciatic nerve sections as a box-whisker plot demonstrating the median values and analyzed by Kruskal–Wallis ANOVA. * *p* < 0.05, and **** *p* < 0.0001, versus the saline group; # *p* < 0.05 versus the DM group.

The histopathological examination of sciatic specimens showed normal multiple axonal sections surrounded by thick myelin sheathes. In addition, the endoneurium appeared with multiple nuclei of Schwann cells (Figure 5A). The diabetic mice showed several axonal and myelin sheath losses and fewer Schwann cell nuclei (Figure 5B). However, mice from the DM+memantine group showed fewer degenerated neuronal axons and an increased number of Schwann cell nuclei (Figure 5C). Panel D demonstrates the scoring for the sciatic specimens and indicated significantly greater scores in the DM group compared to the saline group. Importantly, the registered scores in the DM+memantine group were lower than those registered in the DM control group (Figure 5D).

**Figure 6:**
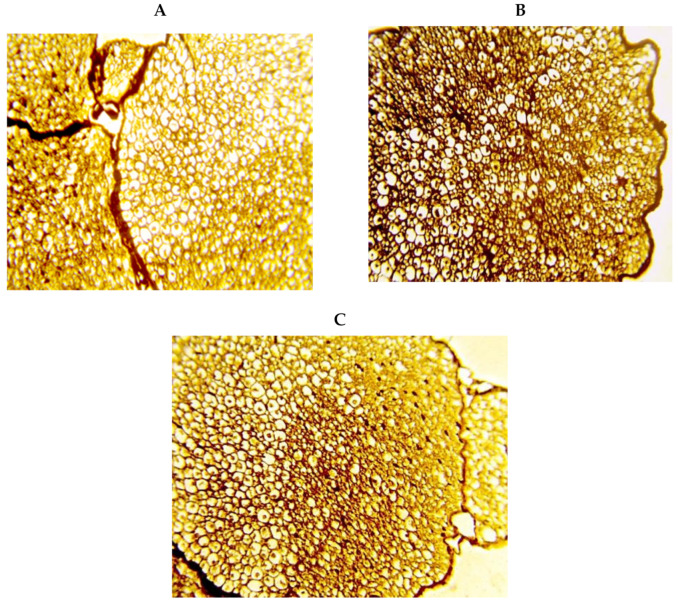
Silver staining for transverse sciatic nerve specimens. Images show well organized nerve fiber axons, including well-structured myelin sheathes enclosed by endoneurium in the saline group (**A**), profound staining by silver stain and a substantial decrease in the myelin sheathes in the diabetic group (**B**), less degeneration in the myelin sheathes and reduced silver staining in the DM+memantine group (**C**). Silver stain ×400.

Silver staining for sciatic cross sections demonstrated well-organized normal nerve fiber axons and well-structured myelin sheathes in the saline group (Figure 6A). Intense silver staining was observed in the DM group accompanied by a substantial decrease in the myelin sheathes (Figure 6B). The DM+memantine group showed noticeably less degeneration (Figure 6C) compared to the DM group.

**Figure 7:**
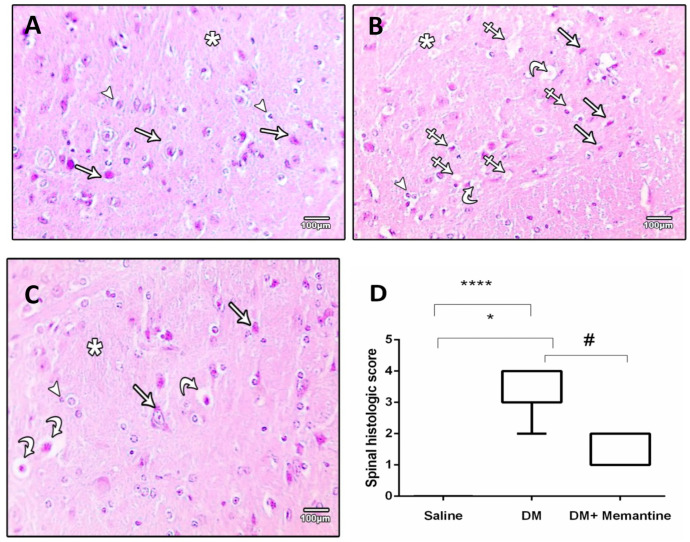
Microscopic pictures of hematoxylin and eosin stained cross sections of spinal cord. (**A**) Saline group showing normal neural cells (arrows) and glia cells (arrow heads) surrounded by neuropils (star). (**B**) Diabetic group (DM) showing marked neural cell degeneration (arrows), vacuoles in the neuropils (crossed arrow), dilated capillaries (curved arrows), and gliosis (arrow head). (**C**) DM+memantine group showing preserved neural cells (arrows) and glial cells (arrow head) with reduced vacuolation in the neuropil (curved arrows). ×100; bar 100 µm. (**D**) box-plot demonstrating the medians of the histopathologic scores assigned for the spinal cord specimens. Analysis was done using Kruskal–Wallis ANOVA. * *p* < 0.05, and **** *p* < 0.0001 versus the saline group; # *p* < 0.05 versus the DM group.

In Figure 7, the spinal specimens stained with hematoxylin and eosin stains showed different pictures in the experimental groups. The saline group showed normally structured neurons (Figure 7A). Specimens from the DM group demonstrated neural degeneration, vacuolation, gliosis, and dilated capillaries (Figure 7B). The DM+memantine group showed preserved spinal neurons (Figure 7C). The spinal scoring demonstrated a greater median score in the DM control group, which was significantly reduced after treatment with memantine (Figure 7D).

**Figure 8:**
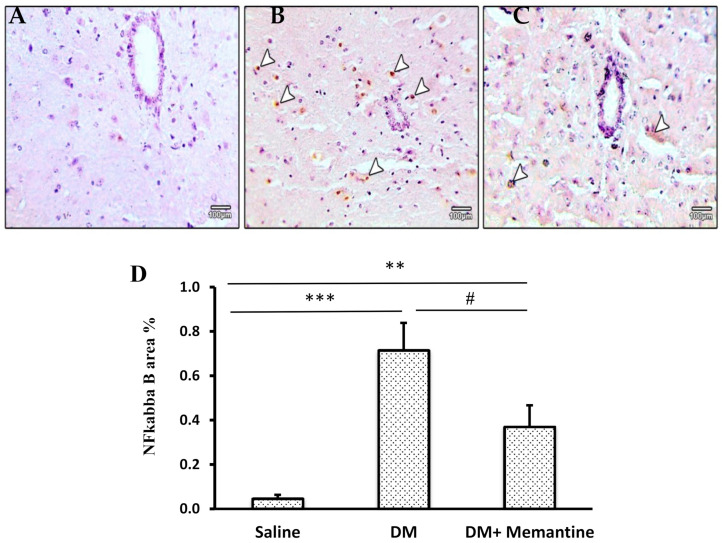
Microscopic pictures of NF-kB immunostained sections from the spinal cords. Images show cross sections with: (**A**) very mild staining in the saline group, (**B**) strong positive nuclear expression in neural cells (arrow heads) from the diabetic group, and (**C**) decreased positive nuclear expression in neural cells (arrow heads) from the DM+memantine group. Immunohistochemistry counterstained with Mayer’s hematoxylin. ×100; bar 100 μm. (**D**) Data are mean staining area ± SDM. DM: diabetes mellitus, SDM: standard deviation of the mean, and NF-kB: nuclear factor-kappa B. ** *p* < 0.01, and *** *p* < 0.001 versus the saline group; # *p* < 0.05 versus the DM group.

Spinal immunohistochemical staining for NF-kB in the saline group was very mild (Figure 8A); however, the DM group showed strong nuclear staining (Figure 8B). The DM+memantine group showed decreased nuclear staining (Figure 8C). The NF-kB stained area in the DM group was about 14-fold greater than in the saline group; this elevated value was reduced in the mice group treated with memantine (Figure 8D).

**Figure 9:**
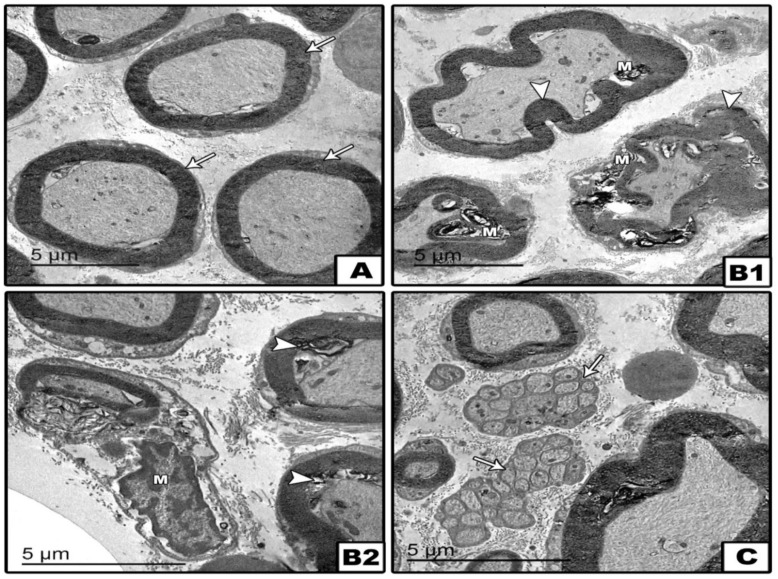
Transmission electron microscopy images of sections of the spinal cord. Images in groups; control, diabetic, and treated diabetic group. Panel (**A**): control section showing the normal characteristics of myelinated axons with regular myelin sheathes (arrows). Panel (**B1**,**B2**) diabetic sections showing degenerated myelinated axons (head arrows) and phagocytic microglia/macrophage cells (M) with degenerating myelin and phagocytosed matter in the cytoplasm in the white matter. The degenerated axons showing disorganized myelin sheathes with swollen vacuolated axoplasm. Microglia are surrounding the degenerated axon of a collapsed myelin sheath. Panel (**C**) Treated group showing myelinated axons with regular myelin sheathes, and very small unmyelinated axons were found with thickened Schwan cell basement membranes (arrows).

Transmission electron microscopy of the spinal cord indicated normal characteristics of myelinated axons with regular myelin sheath in the saline group (Figure 9A). Sections from the DM group showed degenerated myelinated axons and phagocytic microglia/macrophage cells with degenerating myelin and phagocytosed matter in its cytoplasm in white matter. In addition, microglia were found surrounding the degenerated axon of the collapsed myelin sheath (Figure 9B1,B2). The DM+memantine group showed myelinated axons with regular myelin sheathe and very small unmyelinated axons with thickened Schwan cell basement membranes (Figure 9C).

## 3. Discussion

DNP is the most common cause of neuropathy worldwide. It is estimated to affect approximately half of diabetic patients, with a prevalence directly proportionate to the duration of diabetes [21]. DNP is a key therapeutic challenge in spite of the significant advances in understanding the causative mechanisms [5]. Through a well-established alloxan mediated diabetic model with painful neuropathy, we validated the potential role of memantine on the key cellular and molecular pathways involved in the neuroplasticity in the central nociceptive networks, with special shedding on the spinal neuronal–glial interactions, through targeting the HMGB1/TLR4/NF-kB axis and glutamate synaptic transmission.

The persistent hyperglycemia in diabetes represents the most striking and the central cue in the diabetic milieu, precipitating patients to the debilitating DNP [21]. Evidence validated the interneuron dysfunction to contribute critically to the overall altered balance between descending inhibitions and excitation leading to dominance of the descending pain facilitation state, which is responsible for the concomitant hyperalgesia or allodynia [6].

In the current study, alloxan induced apparent allodynia and hyperalgesia as mirrored by a significant reduction in the threshold in the von Frey test [22] and the latencies scored in the hot-plate apparatus. These results matched the previous reports obtained from alloxan-diabetic mice with DNP [23,24].

The treatment of diabetic mice with memantine significantly inhibited allodynia and hyperalgesia reflecting the critical contribution of the NMDARs in the exaggerated pain behavior observed in DNP. Consistently, memantine was reported to suppress the Ca2+ currents and modulate the synaptic plasticity induced by exaggerated glutamate release [25], leading to an evident anti-nociceptive effect [26].

In agreement, clinical studies revealed that memantine reduced DNP in patients [16] but did not explain the exact mechanism. Other NMDA receptor antagonists are obviously successful in regulating chronic pain in patients [27,28,29]. Memantine showed analgesic and neuroprotective effects when used along with pregabalin for treating patients with fibromyalgia syndrome [30].

The elevated spinal glutamate level observed in the diabetic mice in the current study can be attributed primarily to the enhanced glutamate release. Glutamate has been proven to be positively regulated by proinflammatory cytokines that could alter spinal nociceptive processing under DNP [31]. In diabetic conditions, an excessive glutamate level is further exacerbated by reduced glutamate uptake and, consequently, its accumulation in synaptic clefts, secondary to downregulations of the glutamate transporters (GLT-1 and GLAST) in spinal cord astrocytes [25].

The deficient glutamate uptake enhances α-amino-3-hydroxy-5-methyl-4-isoxazolepropionic acid (AMPA) and NMDAR activation, facilitating the glutamate-mediated transcriptional cascades, resulting in an exaggerated response of the dorsal horn cells (DHCs) to tactile and thermal stimuli. This deficient uptake causes glutamate to be spilled to the extrasynaptic space and the activation of extrasynaptic NMDARs in spinal sensory neurons [31]. A low level of NMDAR expression was observed in the saline group and may be explained by the fact that NMDARs contribute little to the fast excitatory transmission, under basal normal conditions [32]. Additionally, the activation of glutamate metabotropic receptors induces the activation of intracellular downstream pathways including protein kinase A, protein kinase C, and mitogen-activated protein kinase pathways that initiate transcription factor activation, including NF*-k*B and cAMP response element-binding protein [33].

The improved hyperalgesia and allodynia in the memantine treated group agrees with prior results that pointed out that deleting NMDAR1-dependent neuronal signaling in rodents defends against the early complete Freund’s adjuvant-induced intraplantar inflammatory allodynia [34] and attenuates formalin-prompted pain behaviors [35]. Interestingly, memantine’s mediated NMDAR blocking effect increases proportionately with enhancing extracellular glutamate levels [36].

A previous paper reported that memantine’s systemic therapy had a strong inhibitory action on rat spinal dorsal horn neuron hypersensitivity prompted through spinal nerve ligation [37]. Intrathecal memantine induced an anti-allodynic action in rats exposed to formalin injection or spinal nerve ligation [38]. Consistently, the topical application of memantine the response of spinothalamic tract cells towards cutaneous stimulation in a dose-dependent manner [39].

The diabetes-induced HMGB1 upregulation is primarily evoked by diabetic oxidative stress, which modulates its degree of activity and HMGB1 translocation from the nucleus to the cytoplasm or extracellular compartment [40]. TLR4 mediates HMGB1 pro-inflammatory effects through the activation of NF-kB. HMGB1/TLR4 initiates a network of downstream cascades with the phosphorylation of many proteins. This positive feedback cycle contributes to the further release of pro-inflammatory cytokines [41,42].

Inflammatory cytokines and oxidative stress can activate NF-kB. Once activated, NF-kB augments the transcription of several inflammatory cytokines, which, in turn, feedback to stimulate NF-kB, creating a positive feedback loop that is supposed to exacerbate the inflammatory signaling, triggering and maintaining ongoing amplified pain signals [43].

Additionally, previous studies indicated that the released HMGB1 activated neuronal TLR4 that is co-localized with NMDARs, resulted in a dramatic onset of post-translational events leading to NMDAR phosphorylation potentiating NMDA-mediated Ca2+ influx, and thus exerting neuromodulatory effects [11,40]. Hence, HMGB1 appears to have an enhancing effect on nociceptive transmission at the spinal dorsal horn neuronal level by enhancing the excitatory neurotransmission and activating astrocytes [7].

Recently, the HMGB1/TLR4 expression levels were reported to be up regulated in spinal neurons of type 2 diabetic rodents with painful neuropathy along with marked mechanical and thermal hyperalgesia [41]. The diabetes-induced HMGB1 shuttling from the nucleus with subsequent activation was triggered mainly by the persistent hyperglycemia and the inflammatory agents, such as IL-1β and TNF-α [41]. Expectedly, interruption of HMGB1-mediated inflammation by a natural HMGB1 inhibitor ameliorated DNP.

G protein-coupled receptors, such as metabotropic glutamate receptors, are known to be triggered by inflammation. Inflammation was proven to upregulate NMDARs through phosphorylation, thereby, increasing the channel open probability and open time. Inflammation prevents NMDARs internalization leading to persistent NMDAR-mediated synaptic currents, thus resulting in pain hypersensitivity [32]. Other evidence implicated IL-1β in NMDAR activation, likely via neutral sphingomyelinase/ceramide, the inositol triphosphate receptor, and phospholipase A2 signaling pathways. Once activated, NMDARs induce a wide-spread glutamate release from neurons, activated astrocytes, and microglia [44].

Under a neuropathic pain state, the released proinflammatory cytokines from activated glial cells, including IL-1β, and TNF-α, are well known to be involved in NP pathogenesis. In presynaptic terminals, IL-1β enhances glutamate release likely through interaction with presynaptic NMDARs eliciting inward Ca2+ currents and is augmented by opening of the voltage-gated Ca2+ channels in response to a depolarization wave leading, subsequently, to glutamate release [31].

The up-regulated expression of proinflammatory molecules reported in the diabetic group could be explained by enhanced release of HMGB1. Recently, researchers [7] pointed to the spinal TLR4 as a HMGB1 target to up-regulate the expression of pronociceptive cytokines, likely through the activation of caspase 1 [31]. In the current study, IL-1β and TNF-α up-regulation was considerably attenuated by memantine therapy. Similarly, intrathecal treatment with IL-1β neutralizing antibodies or the NMDAR antagonist, MK801, effectively prevented HMGB1-induced mechanical hypersensitivity in a dose-dependent manner [7].

Apart from its central action in mitigating glutamatergic transmission, memantine was reported to mitigate NMDARs activation on β-cells, thus abrogating the underlying NF-kB inflammatory/oxidative axis and repressed TNF-*α* and *IL-1β* expression in the pancreas of diabetic mice [43].

The current study acquires some strength points through detailed behavioral and histochemical approaches and that the authors validated the potential of the memantine on the key cellular and molecular pathways involved in the neuroplasticity in the central nociceptive networks. However, some limitations are still present, such as using few animals per group (six mice per group). In addition, we recommend future studies to measure the phosphorylated forms of the target proteins measured to assess the molecular mechanism of memantine.

## 4. Materials and Methods

### 4.1. Animals and Housing

In this experiment, a total of twenty four male Swiss albino mice, with body weights ranging from 20 to 28 g, were utilized. The animals were purchased from the Moustafa Rashed Company (Saqqarah, Egypt). Animal experiments were licensed according to the rules of the Research Ethics Committee (#201906A2a, Faculty of Pharmacy, Suez Canal University). The mice were housed in an air-conditioned atmosphere, at a temperature equal to 25 ± 5 °C with a normal light/dark cycle and regular diet given ad libitum. The mice were acclimatized two weeks before experimentation.

### 4.2. Chemical Agents and Medication

Memantine was a gift from the Tabuk Pharmaceutical Company (Tabuk, Saudi Arabia) and was dissolved in distilled water. Alloxan monohydrate was purchased from SDFCL (Mumbai, India) and dissolved in freshly prepared saline solution.

### 4.3. Inducing Type 1 Diabetes Mellitus in the Male Mice

After an overnight fast, the induction of diabetes was attained by a single intraperitoneal injection of alloxan (180 mg per kg) [45]; this dose was previously reported to induce neuropathy in mice after nine weeks of the single alloxan injection [22,24]. In the sixth day after the injection of alloxan, the fasting blood glucose (FBG) was measured using a One Touch Ultra Mini glucometer (USA) by applying a droplet of blood taken from the tail veins. The mice that had an FBG level greater than 200 mg/dL were counted as diabetic [46].

### 4.4. Experimental Design

Six mice received one saline injection (parallel to alloxan) and served as a vehicle control group. Twelve mice were injected with alloxan to prompt type 1 diabetes mellitus. In the ninth week after the confirmation of hyperglycemia, the development of symptoms of DNP was tested. Briefly, thermal hyperalgesia and mechanical allodynia were measured using the hot-plate test and von Frey filaments, respectively. Diabetic mice showed significant changes in response to thermal and mechanical painful stimuli and were randomly distributed into two groups of mice with six mice each—the diabetic (DM) group and DM+memantine (10 mg/kg)—daily for 35 days [47]. Another group of six healthy mice were injected with saline and given oral memantine (10 mg/kg) starting from the start of week 10 of the experiment and sustained for five weeks. The data from this group are not included in the illustrations.

### 4.5. Behavioral Assessment of Pain Responses

To examine the effect of memantine on DNP-related pain behaviors, the mice were submitted for examination of thermal hyperalgesia and mechanical allodynia.

#### 4.5.1. Measurement of Thermal Hyperalgesia

For the assessment of heat hyperalgesia, a Lsi LETI-CA hot-plate (LE 7406, Italy) was used. The mice were put in a transparent cylinder (diameter = 20 cm and height = 25 cm). Following a 30-min adaption period, each mouse was exposed to radiated heat from the hot-plate that directly affects the hind paw plantar medial surface. To prevent tissue damage, a cut-off time was adjusted at 45 s [48,49]. The hot-plate temperature was set at 55 °C. The latency to licking was determined in terms of the time calculated from positioning the animal on the hot-plate surface until it started paw licking, whereas the latency to jumping was defined as the time to successfully jump off the glass cylinder.

#### 4.5.2. Measurement of Mechanical Allodynia

von Frey filaments were employed for this purpose using the up and down method [50], with slight modifications. The mice were acclimatized for 50 min to the testing environment. An individual plastic cage on a wire-mesh platform was used to permit admission of the hid paw ventral surface. The filaments series (size range is 1.65 to 6.65) was applied in a perpendicular position to the medial surface of the hind paws, starting with the smallest one and with a gradual increase in the tested filaments until the mouse licked or withdrew its paw. The smallest filament size that was able to produce a pain response was taken as the nociceptive threshold and each filament was tested three times per paw and the mean of the three threshold values for each hind paw was calculated for statistical analysis [51].

### 4.6. Sacrification of Mice and Organ Dissection

At the end, the mice were fasted overnight, and the blood glucose level was remeasured. The mice were anesthetized with an intraperitoneal injection of 100/10 mg/kg of ketamine/xylazine mixture [52]. After that, sacrification was done by cervical dislocation, and then a laparotomy was performed to isolate the complete vertebral column. In addition, the sciatic nerves were isolated. In detail, one spinal specimen was taken from the cervical spinal cord and kept at −80 °C for the evaluation of glutamate, TNF-α, and IL-1β as well as the Western blot assays. The whole vertebral column and sciatic nerves were perfused in phosphate-buffered formalin (PBF).

### 4.7. Histopathological and Immunohistochemical Assays

Formalin-fixed sciatic nerves and the cervical spinal cords were embedded in paraffin wax, and then sections of 4 μm were prepared and stained with hematoxylin and eosin (H&E). The slides were evaluated for histopathological alterations by a qualified pathologist in a blind manner. At least three sections were studied from each tissue specimen. The severity of axonopathy, myelinopathy, and sciatic nerve fiber degeneration were assessed and classified as (0–4): (no change, mild, moderate, severe, and very severe) [22,53]. In addition, spinal cord specimens were scored as (0–5): (absent, mild, moderate, severe, very severe, and extremely severe) [23,49], based on the existence of eosinophilic foci of degenerated neurons, vacuolation, and the degree of gliosis in the white and the gray matters [54].

In addition, another section from the sciatic nerve was stained with silver stain to assess the morphology and integrity of the axons and myelin sheaths of sciatic nerve sections; silver staining was performed as previously described [23]. Briefly, sciatic nerve sections were deparaffinized and rinsed in distilled water three times for 5 min each. The sections were impregnated in 10% AgNO_3_ at 37 °C in the dark twice, the first time for 30 min and the second time after washing and adding concentrated ammonium hydroxide for 15 min.

The sections were rinsed in 0.1% NH_4_OH solution and immersed in a coplin jar for 10 min in a developing solution (10 mL distilled water, 0.2 mL of 37% formaldehyde, 0.05 g of citric acid, and 12.5 μL of 20% nitric acid). After a short immersion, the sections were stained with black and were then rinsed in 0.1% NH_4_OH and put in 0.2% AgCl_2_. The sections were then subjected to fixation in 5% Na_2_S_2_O_3_, rinsed in distilled H_2_O, subjected to ascending ethanol concentrations (2 min each) for dehydration and then xylene before mounting.

### 4.8. Immunohistochemical Assessment of NF-kB Protein

The expression of NF-kB in spinal cord specimens was assessed immunohistochemically. Spinal cord tissue sections were incubated with the primary rabbit polyclonal antibody to mouse NF-kB/p65 (ThermoScientific, Cat #RB-1638-R7, Fremont, CA 94538, USA) in a humidity chamber. Then, three fields from each slide were examined and imaged at 400X, and the percentage of immunopositive areas was determined using ImageJ 1.45 F (NIH, USA).

### 4.9. Electron Microscopy

The spinal specimens were fixed in 2.5% glutaraldehyde buffer and then in osmium tetroxide. After that, the specimens were washed and dehydrated in alcohol. They were then inserted in epoxy resin to prepare blocks that were later used for cutting ultra-thin sections, which were subjected to staining by uranyl acetate and lead citrate. Photographs were captured by a JEOL 2100 transmission electron microscope [Tokyo, Japan] [55,56].

### 4.10. Assessment of Spinal Glutamate and Cytokine Levels

The levels of glutamate, TNF-α, and IL-1β in the spinal lysates were estimated using enzyme-linked immunosorbent assay (ELISA) kits. Kits for glutamate (Abanova, cat# KA1909), TNF-α (cat# MBS2507393, My BioSource, Inc., San Diego, CA, USA), and IL-1β (cat# E-EL-R0012c, Elabscience, Houston, TX, USA) were used, and the reaction products were measured colorimetrically.

### 4.11. Western Blotting Analysis for Spinal NMDAR1, HMGB1, TLR4, and p-NF-kB

The spinal tissues were subjected to homogenization in a RIPA buffer solution to which protease and phosphatase inhibitors were added. To remove the insoluble substances, the homogenates were subjected to centrifugation at 12,000× *g* for 25 min. Then, 5 µL of the supernatant was utilized for assessment of the concentration of the protein using a Bio-Rad Quick StartTM Bradford protein assay kit).

Proteins from the tissue homogenates were subjected to a denaturation step using 4X Bio-Rad Laemmli sample buffer and then loaded on sodium dodecyl sulfate polyacrylamide gel. Then, the proteins were electrophoresed and transferred from the gel to nitrocellulose membranes. In order to block the membrane’s free sites, it was incubated in 5% Bio-Rad non-fat dried milk for an hour and finally washed before overnight incubation with the selected primary antibodies: NMDAR1 (NB300-118, NovusBio, USA), HMGB1 (sc-56698, SantaCruz Biotechnology Inc.(Dallas, TX, USA)), TLR4 (sc-293072, SantaCruz Biotechnology Inc.), and p-NF-kB (TA325720, Origene, MD, USA) at 4 °C with gentle agitation.

The following step was the washing of the blots and incubation with horseradish peroxidase–conjugated secondary antibodies [#ab6721] and [#ab205719]. The reacted antigens were visualized by enhanced chemiluminescence by a commercial detection kit (AmershamBioSciences, Buckinghamshire, UK). The densitometric analysis for the color intensity was measured by ImageJ software (NIH) [57].

### 4.12. Statistical Analysis

Data with a Gaussian distribution were processed for analysis with one-way ANOVA and Tukey’s test. These parametric data were demonstrated as the mean± the standard deviation of the mean (SDM). The scoring data were analyzed by non-parametric ANOVA and Dunn’s test. Outlier values were detected by visualizing the data and treated by calculating the mean/median and random imputation in the data pool. All comparisons were done at P values less than 0.05. The saline+memantine group was compared to the saline control group with the unpaired Student’s t test for excluding the per se effect for memantine on pain tests and inflammatory markers.

## 5. Conclusions

Collectively, the current findings documented that glutamate and pro-inflammatory cytokines released in the spinal cord and were the culprits in DNP pathogenesis, through several positive feedback loops. Those loops appeared to be established and maintained through persistent over-activation of the spinal HMGB1/TLR4/NF-kB axis. Memantine effectively disrupted the upstream inflammatory cascade and the downstream signaling pathways from NMDA glutamate receptors and suppressed the spinal HMGB1/TLR4/NF-kB signaling. Therefore, the current study provides further understanding regarding the mechanism of memantine in alleviating DNP and further support for the utility of memantine in diabetic patients.

## Data Availability

Data are available upon request.

## References

[1] Ochoa J.L. (2009). Neuropathic pain: Redefinition and a grading system for clinical and research purposes. Neurology.

[2] Bril V., England J., Franklin G.M., Backonja M., Cohen J., Del Toro D., Feldman E., Iverson D.J., Perkins B., Russell J.W. (2011). Evidence-based Guideline: Treatment of Painful Diabetic Neuropathy: Report of the American Academy of Neurology, the American Association of Neuromuscular and Electrodiagnostic Medicine, and the American Academy of Physical Medicine and Rehabilitation. PMR.

[3] Brod M., Pohlman B., Blum S.I., Ramasamy A., Carson R. (2014). Burden of Illness of Diabetic Peripheral Neuropathic Pain: A Qualitative Study. Patient Patient-Cent. Outcomes Res..

[4] Obrosova I.G. (2009). Diabetic painful and insensate neuropathy: Pathogenesis and potential treatments. Neurotherapeutics.

[5] Rosenberger D.C., Blechschmidt V., Timmerman H., Wolff A., Treede R.-D. (2020). Challenges of neuropathic pain: Focus on diabetic neuropathy. J. Neural Transm..

[6] Colloca L., Ludman T., Bouhassira D., Baron R., Dickenson A.H., Yarnitsky D., Freeman R., Truini A., Attal N., Finnerup N.B. (2017). Neuropathic pain. Nat. Rev. Dis. Prim..

[7] Morioka N., Miyauchi K., Miyashita K., Kochi T., Zhang F.F., Nakamura Y., Liu K., Wake H., Hisaoka-Nakashima K., Nishibori M. (2019). Spinal high-mobility group box-1 induces long-lasting mechanical hypersensitivity through the toll-like receptor 4 and upregulation of interleukin-1β in activated astrocytes. J. Neurochem..

[8] Maeda T., Ozaki M., Kobayashi Y., Kiguchi N., Kishioka S. (2013). HMGB1 as a Potential Therapeutic Target for Neuropathic Pain. J. Pharmacol. Sci..

[9] Pedrazzi M., Averna M., Sparatore B., Patrone M., Salamino F., Marcoli M., Maura G., Cervetto C., Frattaroli D., Pontremoli S. (2012). Potentiation of NMDA Receptor-Dependent Cell Responses by Extracellular High Mobility Group Box 1 Protein. PLoS ONE.

[10] Wan W., Cao L., Khanabdali R., Kalionis B., Tai X., Xia S. (2016). The Emerging Role of HMGB1 in Neuropathic Pain: A Potential Therapeutic Target for Neuroinflammation. J. Immunol. Res..

[11] Balosso S., Liu J., Bianchi M.E., Vezzani A. (2014). Disulfide-Containing High Mobility Group Box-1 PromotesN-Methyl-d-Aspartate Receptor Function and Excitotoxicity by Activating Toll-Like Receptor 4-Dependent Signaling in Hippocampal Neurons. Antioxid. Redox Signal..

[12] Kishi T., Matsunaga S., Oya K., Nomura I., Ikuta T., Iwata N. (2017). Memantine for Alzheimer’s Disease: An Updated Systematic Review and Meta-analysis. J. Alzheimer’s Dis..

[13] Rajasekar N., Nath C., Hanif K., Shukla R. (2015). Inhibitory Effect of Memantine on Streptozotocin-Induced Insulin Receptor Dysfunction, Neuroinflammation, Amyloidogenesis, and Neurotrophic Factor Decline in Astrocytes. Mol. Neurobiol..

[14] Cheng Q., Fang L., Feng D., Tang S., Yue S., Huang Y., Han J., Lan J., Liu W., Gao L. (2019). Memantine ameliorates pulmonary inflammation in a mice model of COPD induced by cigarette smoke combined with LPS. Biomed. Pharmacother..

[15] Sevostianova N., Danysz W., Bespalov A.Y. (2005). Analgesic effects of morphine and loperamide in the rat formalin test: Interactions with NMDA receptor antagonists. Eur. J. Pharmacol..

[16] Sang C.N., Booher S., Gilron I., Parada S., Max M.B. (2002). Dextromethorphan and Memantine in Painful Diabetic Neuropathy and Postherpetic NeuralgiaEfficacy and Dose-Response Trials. J. Am. Soc. Anesthesiol..

[17] Rogers M., Rasheed A., Moradimehr A., Baumrucker S.J. (2008). Memantine (Namenda) for Neuropathic Pain. Am. J. Hosp. Palliat. Med..

[18] Nikolajsen L., Gottrup H., Kristensen A.G., Jensen T.S. (2000). Memantine (a N-methyl-D-aspartate receptor antag-onist) in the treatment of neuropathic pain after amputation or surgery: A randomized, double-blinded, cross-over study. Anesth. Analg..

[19] Fashner J., Bell A.L. (2011). Herpes zoster and postherpetic neuralgia: Prevention and management. Am. Fam. Physician.

[20] McCormick Z., Chang-Chien D.G., Marshall D.B., Huang M., Harden R.N. (2014). Phantom Limb Pain: A Systematic Neuroanatomical-Based Review of Pharmacologic Treatment. Pain Med..

[21] Iqbal Z., Azmi S., Yadav R., Ferdousi M., Kumar M., Cuthbertson D.J., Lim J., Malik R.A., Alam U. (2018). Diabetic Peripheral Neuropathy: Epidemiology, Diagnosis, and Pharmacotherapy. Clin. Ther..

[22] Tawfik M.K., Helmy S.A., Badran D.I., Zaitone S.A. (2018). Neuroprotective effect of duloxetine in a mouse model of diabetic neuropathy: Role of glia suppressing mechanisms. Life Sci..

[23] Elsherbiny N.M., Ahmed E., Kader G.A., Abdel-Mottaleb Y., ElSayed M.H., Youssef A.M., Zaitone S.A. (2019). In-hibitory effect of valproate sodium on pain behavior in diabetic mice involves suppression of spinal histone deacetylase 1 and inflammatory mediators. Int. Immunopharmacol..

[24] El-Sherbeeny N.A., Ibrahiem A.T., Ali H.S., Farag N.E., Toraih E.A., Zaitone S.A. (2020). Carbamazepine conquers spinal GAP43 deficiency and sciatic Nav1.5 upregulation in diabetic mice: Novel mechanisms in alleviating allodynia and hyperalgesia. Arch. Pharmacal Res..

[25] Ji P.R.-R., Nackley P.A., Huh B.Y., Terrando P.N., Maixner D.W. (2018). Neuroinflammation and Central Sensitization in Chronic and Widespread Pain. Anesthesiology.

[26] Chen S.-R., Samoriski G., Pan H.-L. (2009). Antinociceptive effects of chronic administration of uncompetitive NMDA receptor antagonists in a rat model of diabetic neuropathic pain. Neuropharmacology.

[27] Correll G.E., Maleki J., Gracely E.J., Muir J.J., Harbut R.E. (2004). Subanesthetic ketamine infusion therapy: A ret-rospective analysis of a novel therapeutic approach to complex regional pain syndrome. Pain Med..

[28] Cvrček P. (2008). Side Effects of Ketamine in the Long-Term Treatment of Neuropathic Pain. Pain Med..

[29] Fisher K., Coderre T.J., Hagen N.A. (2000). Targeting the N-methyl-D-aspartate receptor for chronic pain manage-ment: Preclinical animal studies, recent clinical experience and future research directions. J. Pain Symptom Manag..

[30] Recla J.M., Sarantopoulos C.D. (2009). Combined use of pregabalin and memantine in fibromyalgia syndrome treatment: A novel analgesic and neuroprotective strategy?. Med. Hypotheses.

[31] Yan X., Weng H.-R. (2013). Endogenous Interleukin-1β in Neuropathic Rats Enhances Glutamate Release from the Primary Afferents in the Spinal Dorsal Horn through Coupling with Presynaptic N-Methyl-d-aspartic Acid Receptors. J. Biol. Chem..

[32] Liu X.J., Salter M.W. (2010). Glutamate receptor phosphorylation and trafficking in pain plasticity in spinal cord dorsal horn. Eur. J. Neurosci..

[33] Gwak Y.S., Hulsebosch C.E., Leem J.W. (2017). Neuronal-Glial Interactions Maintain Chronic Neuropathic Pain after Spinal Cord Injury. Neural Plast..

[34] Weyerbacher A.R., Xu Q., Tamasdan C., Shin S.J., Inturrisi C.E. (2010). N -Methyl-d-aspartate receptor (NMDAR) independent maintenance of inflammatory pain. Pain.

[35] Garraway S.M., Xu Q., Inturrisi C.E. (2009). siRNA-Mediated Knockdown of the NR1 Subunit Gene of the NMDA Receptor Attenuates Formalin-Induced Pain Behaviors in Adult Rats. J. Pain.

[36] Olivares D., Deshpande V.K., Shi Y., Lahiri D.K., Greig N.H., Rogers J.T., Huang X. (2012). N-Methyl D-Aspartate (NMDA) Receptor Antagonists and Memantine Treatment for Alzheimer’s Disease, Vascular Dementia and Parkinson’s Disease. Curr. Alzheimer Res..

[37] Suzuki R., Matthews E.A., Dickenson A.H. (2001). Comparison of the effects of MK-801, ketamine and memantine on responses of spinal dorsal horn neurones in a rat model of mononeuropathy. Pain.

[38] Chaplan S.R., Malmberg A.B., Yaksh T.L. (1997). Efficacy of spinal NMDA receptor antagonism in formalin hyper-algesia and nerve injury evoked allodynia in the rat. J. Pharmacol. Exp. Ther..

[39] Carlton S.M., Rees H., Tsuruoka M., Willis W.D. (1998). Memantine attenuates responses of spinothalamic tract cells to cutaneous stimulation in neuropathic monkeys. Eur. J. Pain.

[40] Paudel Y.N., Semple B.D., Jones N.C., Othman I., Shaikh M.F. (2019). High mobility group box 1 (HMGB1) as a novel frontier in epileptogenesis: From pathogenesis to therapeutic approaches. J. Neurochem..

[41] Thakur V., Sadanandan J., Chattopadhyay M. (2020). High-Mobility Group Box 1 Protein Signaling in Painful Diabetic Neuropathy. Int. J. Mol. Sci..

[42] Elshaer R.E., Tawfik M.K., Nosseir N., El-Ghaiesh S.H., Toraih E.A., Elsherbiny N.M., Zaitone S.A. (2019). Leflunomide-induced liver injury in mice: Involvement of TLR4 mediated activation of PI3K/mTOR/NFκB pathway. Life Sci..

[43] Huang X.-T., Li C., Peng X.-P., Guo J., Yue S.-J., Liu W., Zhao F.-Y., Han J.-Z., Huang Y.-H., Yang-Li Y.-L. (2017). An excessive increase in glutamate contributes to glucose-toxicity in β-cells via activation of pancreatic NMDA receptors in rodent diabetes. Sci. Rep..

[44] Sung C.S., Wen Z.H., Feng C.W., Chen C.H., Huang S.Y., Chen N.F., Chen W.F., Wong C.S. (2017). Potentiation of spinal glutamatergic response in the neuron-glia interactions underlies the intrathecal IL-1β-induced thermal hyperal-gesia in rats. CNS Neurosci. Ther..

[45] Kikumoto Y., Sugiyama H., Inoue T., Morinaga H., Takiue K., Kitagawa M., Fukuoka N., Saeki M., Maeshima Y., Wang D.-H. (2010). Sensitization to alloxan-induced diabetes and pancreatic cell apoptosis in acatalasemic mice. Biochim. Biophys. Acta (BBA) Mol. Basis Dis..

[46] Elsherbiny N.M., Abdel-Mottaleb Y., Elkazaz A.Y., Atef H., Lashine R.M., Youssef A.M., Ezzat W., El-Ghaiesh S.H., Elshaer R.E., El-Shafey M. (2019). Carbamazepine Alleviates Retinal and Optic Nerve Neural Degeneration in Diabetic Mice via Nerve Growth Factor-Induced PI3K/Akt/mTOR Activation. Front. Neurosci..

[47] Gao L., Chen X., Tang Y., Zhao J., Li Q., Fan X., Xu H., Yin Z.Q. (2015). Neuroprotective effect of memantine on the retinal ganglion cells of APPswe/PS1ΔE9 mice and its immunomodulatory mechanisms. Exp. Eye Res..

[48] Higgs J., Wasowski C., Loscalzo L.M., Marder M. (2013). In vitro binding affinities of a series of flavonoids for μ-opioid receptors. Antinociceptive effect of the synthetic flavonoid 3,3-dibromoflavanone in mice. Neuropharmacology.

[49] Reda H.M., Zaitone S.A., Moustafa Y.M. (2016). Effect of levetiracetam versus gabapentin on peripheral neuropathy and sciatic degeneration in streptozotocin-diabetic mice: Influence on spinal microglia and astrocytes. Eur. J. Pharmacol..

[50] Chaplan S., Bach F., Pogrel J., Chung J., Yaksh T. (1994). Quantitative assessment of tactile allodynia in the rat paw. J. Neurosci. Methods.

[51] Yalcin I., Benbouzid M., Tessier L.-H., Müller A., Hein L., Barrot M., Choucair-Jaafar N., Freund-Mercier M.-J. (2009). β2-adrenoceptors are critical for antidepressant treatment of neuropathic pain. Ann. Neurol..

[52] Ko M.J., Mulia G.E., Van Rijn R.M. (2019). Commonly Used Anesthesia/Euthanasia Methods for Brain Collection Differentially Impact MAPK Activity in Male and Female C57BL/6 Mice. Front. Cell. Neurosci..

[53] Greish S., Abogresha N., Zaitone S. (2014). Duloxetine Modulates Vincristine-Induced Painful Neuropathy in Rats. J. Physiol. Pharmacol. Adv..

[54] Eaton S.E., Harris N.D., Rajbhandari S.M., Greenwood P., Wilkinson I.D., Ward J.D., Griffiths P.D., Tesfaye S. (2001). Spinal-cord involvement in diabetic peripheral neuropathy. Lancet.

[55] Reynolds E.S. (1963). The use of lead citrate at high PH as an electron-opaque stain in electron microscopy. J. Cell Biol..

[56] Morris J.K. (1965). A formaldehyde glutaraldehyde fixative of high osmolality for use in electron microscopy. J. Cell Biol..

[57] El-Ghaiesh S.H., Bahr H.I., Ibrahiem A.T., Ghorab D., AlOmar S.Y., Farag N.E., Zaitone S.A. (2020). Metformin Protects from Rotenone–Induced Nigrostriatal Neuronal Death in Adult Mice by Activating AMPK-FOXO3 Signaling and Mitigation of Angiogenesis. Front. Mol. Neurosci..

